# Evolutionary Dynamics and Lateral Gene Transfer in Raphidophyceae Plastid Genomes

**DOI:** 10.3389/fpls.2022.896138

**Published:** 2022-05-26

**Authors:** Jong Im Kim, Bok Yeon Jo, Myung Gil Park, Yeong Du Yoo, Woongghi Shin, John M. Archibald

**Affiliations:** ^1^Department of Biology, Chungnam National University, Daejeon, South Korea; ^2^Nakdonggang National Institute of Biological Resources, Sangju, South Korea; ^3^LOHABE, Department of Oceanography, Chonnam National University, Gwangju, South Korea; ^4^Department of Marine Biology, College of Ocean Sciences and Technology, Kunsan National University, Kunsan, South Korea; ^5^Department of Biochemistry and Molecular Biology, Dalhousie University, Halifax, NS, Canada

**Keywords:** genome reduction, lateral gene transfer, mosaicism, plastid genome, Raphidophyceae

## Abstract

The Raphidophyceae is an ecologically important eukaryotic lineage of primary producers and predators that inhabit marine and freshwater environments worldwide. These organisms are of great evolutionary interest because their plastids are the product of eukaryote-eukaryote endosymbiosis. To obtain deeper insight into the evolutionary history of raphidophycean plastids, we sequenced and analyzed the plastid genomes of three freshwater and three marine species. Our comparison of these genomes, together with the previously reported plastid genome of *Heterosigma akashiwo*, revealed unexpected variability in genome structure. Unlike the genomes of other analyzed species, the plastid genome of *Gonyostomum semen* was found to contain only a single rRNA operon, presumably due to the loss of genes from the inverted repeat (IR) region found in most plastid genomes. In contrast, the marine species *Fibrocapsa japonica* contains the largest IR region and overall plastid genome for any raphidophyte examined thus far, mainly due to the presence of four large gene-poor regions and foreign DNA. Two plastid genes, *tyr*C in *F. japonica* and *He. akashiwo* and *ser*C in *F. japonica*, appear to have arisen *via* lateral gene transfer (LGT) from diatoms, and several raphidophyte open reading frames are demonstrably homologous to sequences in diatom plasmids and plastid genomes. A group II intron in the *F. japonica psb*B gene also appears to be derived by LGT. Our results provide important insights into the evolutionary history of raphidophyte plastid genomes *via* LGT from the plastids and plasmid DNAs of diatoms.

## Introduction

The Raphidophyceae, a class of photosynthetic stramenopile algae, is an evolutionarily diverse and ecologically important lineage whose members thrive in both marine and freshwater environments. The common marine genera *Chattonella* and *Heterosigma* are known to be toxic and capable of forming red tides (Hara and Chihara, 1982). Raphidophyte cells are flagellated with two unequal emergent flagella, numerous discoid chloroplasts, and mucocysts (Heywood, 1982, 1990). Three freshwater genera (*Gonyostomum*, *Merotricha*, and *Vacuolaria*) and seven marine genera (*Chattonella*, *Chlorinimonas*, *Fibrocapsa*, *Haramonas, Heterosigma*, *Psammamonas*, *and Viridilobus*) have been formally described (Fott, 1968; Heywood, 1990; Horiguchi, 1996). Most marine members possess fucoxanthin as their main accessory photosynthetic pigment and have yellowish brown-colored plastids. All freshwater species described thus far are green in color due to the absence of fucoxanthin (Fiksdahl et al., 1984; Bjørnland and Liaaen-Jensen, 1989). Little is known about the extent to which differences in habitat and pigment composition have contributed to the evolutionary diversification of raphidophycean algae.

The plastid of photosynthetic stramenopiles stems from rhodophytes *via* secondary or possibly tertiary endosymbiosis and is very different from those of the primary plastid-bearing green algae, land plants, glaucophytes, and rhodophytes. Recent phylogenomic studies of photosynthetic stramenopiles have included plastid genome data from Bacillariophyceae (diatoms), Bolidophyceae, Chrysophyceae, Dictyochophyceae, Eustigmatophyceae, Olisthodiscophyceae, Phaeophyceae (brown algae), Pelagophyceae, Xanthophyceae, and Raphidophyceae, as well as plastid-bearing alveolates (Cattolico et al., 2008; Ong et al., 2010; Ruck et al., 2014; Ševčíková et al., 2015; Han et al., 2019; Kim et al., 2019, 2020; Sassenhagen and Rengefors, 2019; Barcytė et al., 2021). The genus *Olisthodiscus* has recently been transferred from Raphidophyceae to the newly erected class Olisthodiscophyceae based on phylogenomics of plastid sequences (Barcytė et al., 2021).

To better understand the relationships among raphidophycean algae as well as the broader evolution of plastids in stramenopiles, we sequenced six raphidophyte plastid genomes, two of which are from the freshwater species *Gonyostomum semen* and *Vacuolaria virescens*, and carried out detailed comparative genomic and phylogenomic analyses of these data, as well as the published plastid genome of the raphidophycean *Heterosigma akashiwo* [pre- existing but incomplete plastid genomic data for *G. semen* and *V. virescens* were not included (Sassenhagen and Rengefors, 2019)]. We discovered instances of gene loss/gain, duplication/reduction of the inverted repeat (IR) region, and gene rearrangements in raphidophycean plastid genomes. We also show that their genomes have been impacted by lateral gene transfer (LGT) from diatoms.

## Materials and Methods

### DNA Isolation and Sequencing

Strains of *Gonyostomum semen*, *Vacuolaria virescens*, *Chattonella marina*, and *Fibrocapsa japonica* were collected from natural habitats: *G. semen* from freshwater, Geumsan, Chungcheongnam-do, Korea (36° 10′ 53.9” N 127° 27′ 38.4″ E), *V. virescens* from freshwater, Changnyeong, Gyeongsangnam-do, Korea (35° 31′ 15.7” N 128° 23′ 08″ E), *C. marina* from marine, Kunsan, Jeollanam-do, Korea (35° 56′ 11.1’’ N, 126° 31′ 39.4″ E), and *F. japonica* from marine, Masanman, Gyeongsangnam-do, Korea (35° 12′ 02.7” N, 128° 34′ 40.3″ E). The *G. semen* and *V. virescens* strains are available from the culture collection at the Chungnam National University Korea. The *C. marina* and *F. japonica* strains are available from the culture collections at the Kunsan National University or Chonnam National University Korea. Cultured strains of *Merotricha bacillata* NIES-1809 and *Haramonas pauciplastida* NIES-1870 were obtained from the Microbial Culture Collection (MCC), National Institute for Environmental Studies (NIES), Japan. All cultures studied herein were grown in AF-6 or f/2 medium (Andersen et al., 2005) with distilled water for the freshwater strains or distilled seawater for the marine strains and were maintained at 20°C under a 14:10 light:dark cycle with 30 μmol photons·m^−2^·s^−1^ from cool white fluorescent tubes. All strains were derived from single-cell isolation for unialgal cultivation. Total genomic DNAs were extracted using the QIAGEN DNEasy Blood Mini Kit (QIAGEN, Valencia, CA, United States) following the manufacturer’s instructions. Next-generation sequencing was carried out using a MiSeq (Illumina, San Diego, CA, United States). Amplified DNAs were fragmented and tagged using the NexteraXT protocol (Illumina), indexed, size selected, and pooled for sequencing using the small amplicon targeted resequencing run, which performs paired end 2 × 300 bp sequencing reads using the MiSeq Reagent Kit v3 (Illumina), according to the manufacturer’s recommendations.

### Genome Assembly and Annotation of Plastid Genomes

Sequence data were trimmed (base = 80 bp, error threshold = 0.05, n ambiguities = 2) prior to *de novo* assembly with the default option (automatic bubble size, minimum contig length = 1,000 bp). The raw reads were assembled using the SPAdes 3.7 assembler^1^ and mapped to the assembled contigs (similarity = 95%, length fraction = 75%), excluding contigs <1,000 bp (assembly statistics are summarized in Supplementary Table S1). Contigs were deemed to be of plastid genome origin as follows: (1) BLAST searches against the entire assembly using commonly known plastid genes as queries resulted in hits to these contigs using Genome Search Plotter (Ejigu et al., 2021) and (2) the predicted genome sizes were similar to the previously published 160 Kbp plastid genome of *He. akashiwo* NIES-293 (NC_010882).

To aid in gene annotation, we created a database of protein-coding genes, rRNA, and tRNA genes using data from previously sequenced raphidophycean plastid genomes. Preliminary annotation of protein-coding genes was performed using AGORA (Jung et al., 2018) and GeneMarkS.^2^ The final annotation file was checked in Geneious Prime^3^ using the ORF Finder program with genetic code 11 (Bacterial, Archaeal, and Plant Plastid Code). The predicted ORFs were checked manually and the corresponding ORFs (and predicted functional domains) in the genome sequences were annotated accordingly.

The tRNA genes were identified from the tRNAscan-SE version 1.21 server^4^ with the default settings using the “Mito/Chloroplast” model. To help identify rRNA gene sequences, a set of known plastid rRNA sequences from the public database was used as query sequences to search new genomic data using BLASTn. We used RNAweasel^5^ to search for, and classify introns. Physical maps were visualized with the OrganellarGenomeDRAW program.^6^ For structural and synteny comparisons, the genomes were aligned using Mauve Genome Alignment version 2.2.0 (Darling et al., 2004) and geneCo (Jung et al., 2019) with default settings. Genome sequences were deposited in the NCBI GenBank database under the accession numbers shown in Table 1.

**Table 1:** Characteristics of raphidophycean plastid genomes analyzed in this study.

General characteristics	*Gonyostomum semen* KR	*Merotricha bacillata* NIES-1809	*Vacuolaria virescens* KR	*Chattonella marina* KR	*Fibrocapsa japonica* KR	*Haramonas pauciplastida* NIES-1870	*Heterosigma akashiwo* NIES-293
Habitat	Freshwater	Freshwater	Freshwater	Marine	Marine	Marine	Marine
Color	Green	Green	Green	Yellowish brown	Yellowish brown	Yellowish brown	Yellowish brown
Size (bp)	122,284	123,907	137,965	138,736	248,913	177,770	159,370
Inverted repeat (IR)	absent	5,393	9,255	6,166	52,431	36,445	21,665
Small single-copy region	–	33,190	33,866	38,827	15,531	1,418	38,834
Large single-copy region	–	79,931	85,589	87,577	128,520	103,462	76,756
G + C (%)	28	27.5	26.5	31.5	34.8	32.3	31.4
Total genes (inc. RNAs)	170	175	181	182	237	205	198
No. of protein-coding genes	141	142	148	148	199	172	156
tRNAs	27	29	30	30	35	30	35
rRNA operons	1	2	2	2	2	2	2
Unknown ORFs	6	5	13	7	42	11	8
No. of duplicated genes	none	5	7	4	33	37	25
Pseudogenes	*trn*R, *trn*E	none	*trn*E	none	none	none	none
GenBank accession	ON228255	ON228256	ON228257	ON228258	ON228260	ON228259	NC_010882

### Phylogenetic Analysis

A phylogenetic analysis of diverse eukaryotic algae was carried out on a concatenated set of 91 proteins (18,216 amino acid sites in total) encoded in 135 plastid genomes (Supplementary Figure S1). The sequences of six Viridiplantae and one glaucophyte species were used as outgroup taxa for rooting purposes. The concatenated proteins were aligned using MacGDE2.6 with manual refinement (Smith et al., 1994). Alignments for protein-specific phylogenetic analyses were constructed for *tsg*1, *ser*C1/*ser*C2, *tyr*C, the reverse transcriptase domain of group II intron-encoded proteins, and various conserved hypothetical ORFs. For each protein, homologs were retrieved from the NCBI non-redundant database using BLASTp (*e*-value cutoff = 1*e*^−5^) with raphidophyte proteins as queries. ML phylogenetic analyses of individual protein alignments and concatenated alignments were conducted using IQ-TREE Ver. 1.5.2 (Nguyen et al., 2015) with 1,000 bootstrap replicates. The best evolutionary model for each tree was automatically selected using the –m LG + I + G option incorporated in IQ-TREE. Trees were visualized using FigTree v.1.4.2.^7^

## Results and Discussion

### General Features of Raphidophyceae Plastid Genomes

**Figure 1:**
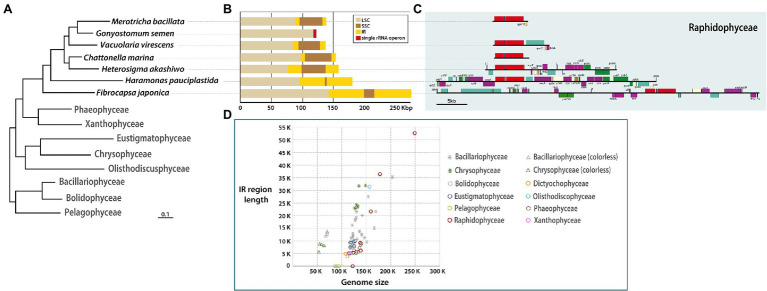
**(A)** Phylogenetic tree based on plastid genes (see Supplementary Figure S1). **(B)** Sequence length of SSC, LSC, IR regions. **(C)** Detailed gene contents in IR region of each raphidophycean species. **(D)** Distribution graph for length of IR region vs. total plastid genome among stramenopiles.

**Figure 2:**
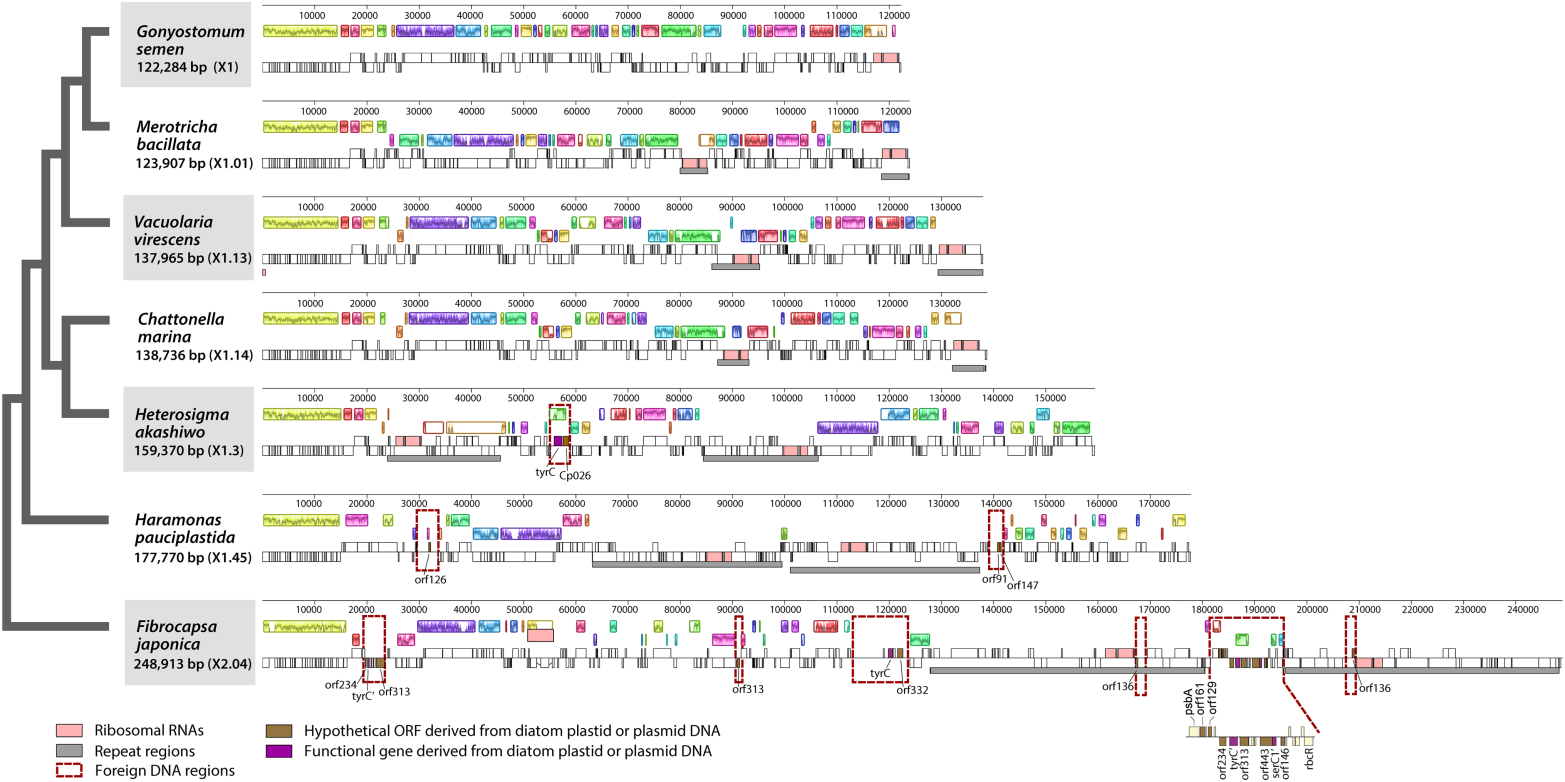
Linearized maps of the plastid genomes of Raphidophyceae. Foreign DNA-containing regions are highlighted with red-dashed lines.

Six plastid genomes (ptDNA) of representative raphidophycean strains from freshwater and marine habitats were sequenced (Table 1). The structure and coding capacity of these ptDNAs were compared to the previously published genome of the raphidophycean alga *He. akashiwo* NIES-293 (Cattolico et al., 2008). Plastid genome sizes ranged from 122 Kbp (*G. semen*) to 249 Kbp (*F. japonica*) with GC content ranging from 26.5% (*V. virescens*) to 34.8% (*F. japonica*). The plastid genomes contained 141 (*G. semen*) to 199 (*F. japonica*) protein-coding genes (including hypothetical proteins), 3 rRNAs, and 27 ~ 35 tRNAs. Minor variation was found in tRNA gene content. For instance, *trn*E^UUC^ is present in *G. semen* and *V. virescens*, and *trn*R^ACG^ is present in *G. semen* as a pseudogene. The raphidophyte plastid genomes generally showed canonical structure in possessing a large single-copy region (LSC), a small single-copy region (SSC), and two inverted repeats (IRs) with protein-coding genes and ribosomal RNA operons. The only exception is *G. semen*, which is highly unusual in possessing a single ribosomal RNA operon (Figures 1, 2; Supplementary Figure S2). As described in the sections that follow, the increase in plastid genome size in some raphidophyte species is mainly caused by the expansion of gene-poor regions and acquisition of foreign sequences from the plastid or plasmid DNAs of diatoms (red-dashed boxes in Figure 2). Remarkably, the foreign sequences in the *F. japonica* plastid genomes make it almost twice the size of the *G. semen* genome.

### Inverted Repeat Expansion, Contraction, and Loss

The newly sequenced raphidophycean plastid genomes show evidence of species-specific gene order and content expansion, contraction, and loss in their IR regions (Figures 1B,C). The IR sequence length was found to range from 0 Kbp (i.e., no repeat region) to 52.43 Kbp with functional protein-coding genes, hypothetical ORFs, three rRNAs, and tRNAs. The gene content in the plastid genome of *G. semen* was distinct from all other raphidophycean algal plastid genomes. The small single-copy (SSC) region varies in length, ranging from 1,418 bp (*Ha. pauciplastida*) to 38,834 bp (*He. akashiwo*; Figures 1B,C).

IR-related plastid genome dynamics is a well-studied phenomenon (Goulding et al., 1996; Wang et al., 2008). Contractions, expansions, and small-scale changes in IR and SSC regions have been documented in the plastid genomes of diatoms, chrysophytes, and green algae, sometimes giving rise to changes in gene content (Jansen and Ruhlman, 2012; Sabir et al., 2014; Turmel et al., 2015; Kim et al., 2019, 2020). Expansions and contractions of the IR region have occurred during the evolutionary history of Raphidophyceae as well, leading to changes in gene content and length (Figures 1, 2; Supplementary Figure S2). The size of the IR region correlates strongly with total plastid genome size among stramenopile lineages. The Bacillariophyceae and Chrysophyceae genomes separate into two clusters because of genome reduction in non-photosynthetic groups (stars and triangles in Figure 1D). The pelagophycean algae studied thus far have only single-copy genes in their plastid genomes (Ong et al., 2010). Interestingly, the raphidophycean algae have both IR-lacking and IR-containing plastid genome types and show the largest plastid genome size variation among stramenopile lineages studied thus far (Figure 1D, red circles).

### Lineage-Specific Gene Loss

**Figure 3:**
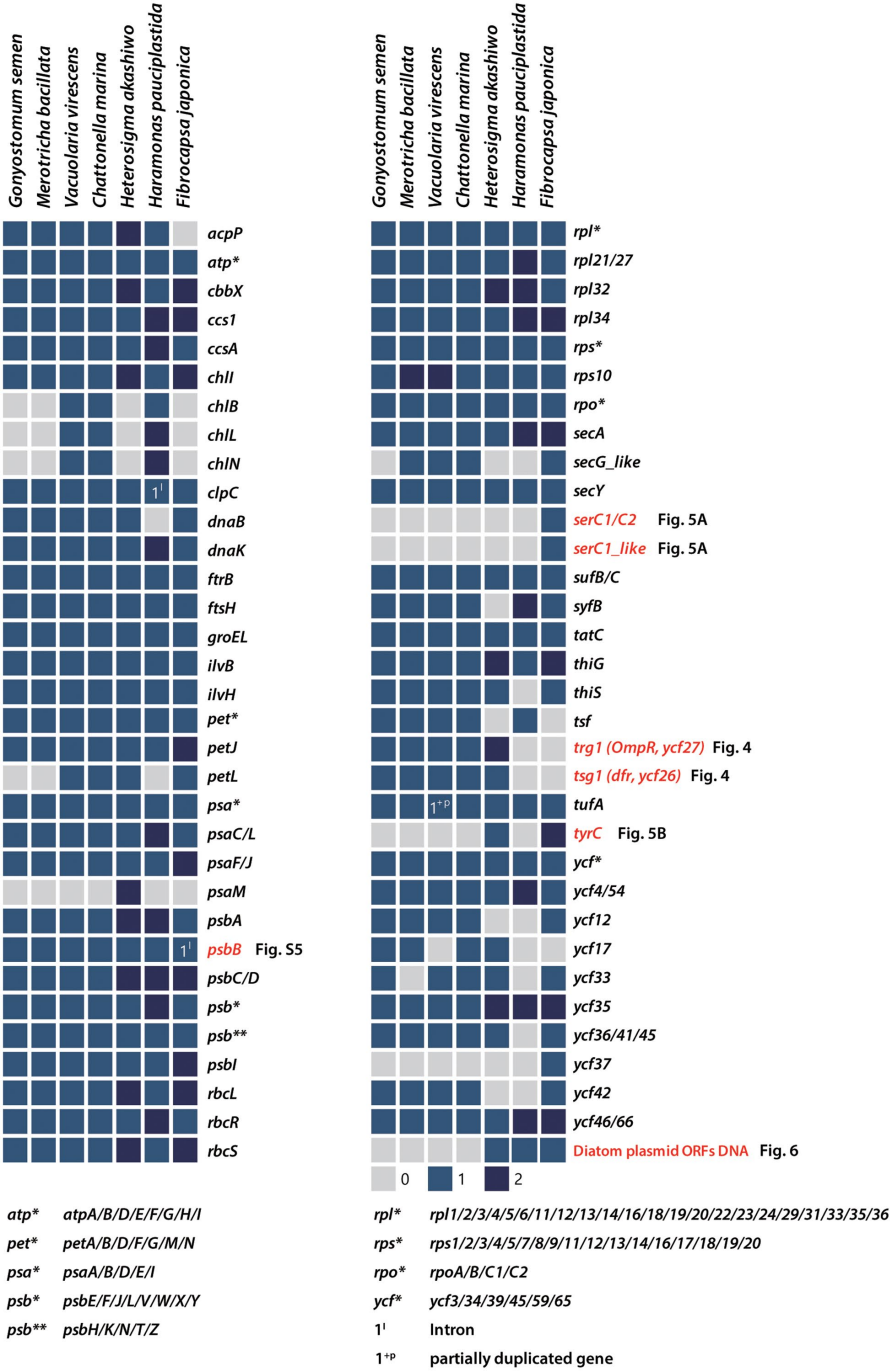
Presence/absence of plastid-encoded genes in Raphidophyceae. Filled boxes indicate the status of each gene (grey = absent, blue = present, dark blue = 2 copies). Patchily distributed genes (i.e., *Ser*C1, *ser*C1-like, *trg*1, *tsg*1, *tyr*C, and various hypothetical ORFs) were detected in the plastid genomes and phylogenies were constructed to infer their evolutionary history.

**Figure 4:**
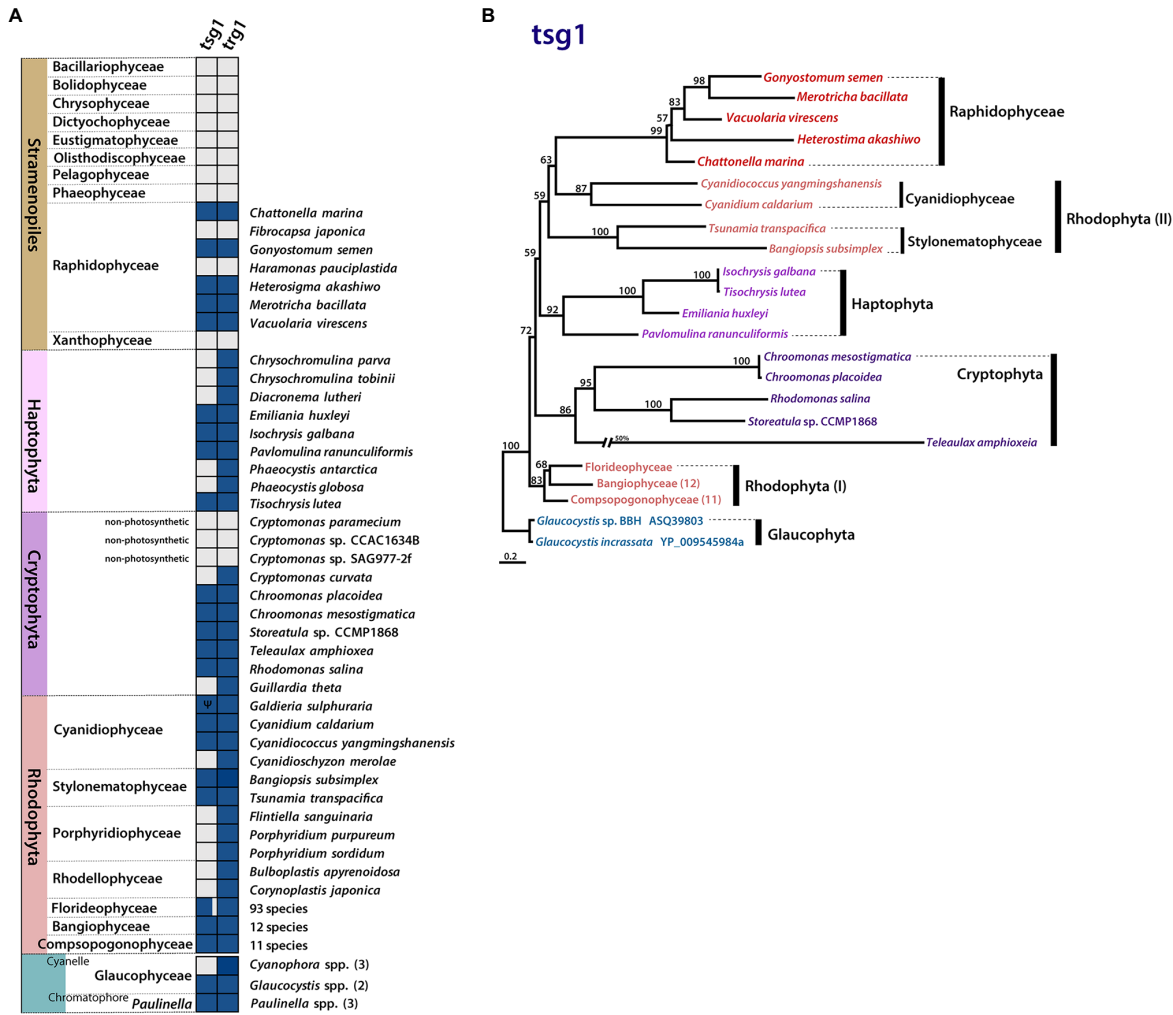
Plastid gene gain and loss in Raphidophyceae. **(A)** Presence/absence of plastid-encoded *tsg*1 and *trg*1 genes. Filled boxes indicate the status of each gene (grey = absent, blue = present, Ψ = pseudogene). **(B)** Phylogenetic tree of *tsg*1 protein sequences in Rhodophyta, red alga-derived lineages, and Glaucophyta. Support values on branches are from IQ-Tree UFBoot. The scale bar shows the inferred number of amino acid substitutions per site.

**Figure 5:**
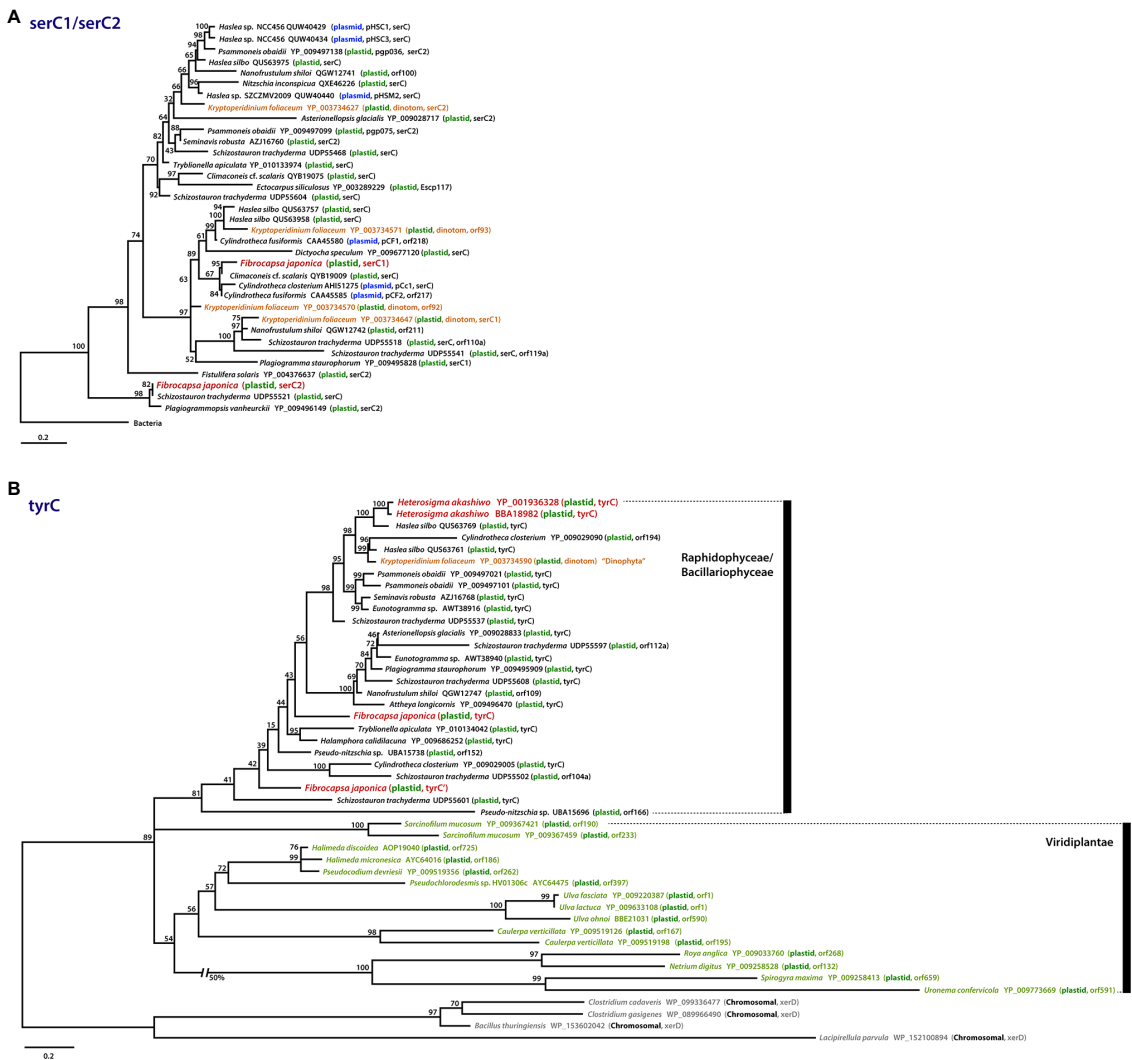
Phylogenies of patchily distributed genes in raphidophyte plastid genomes and their homologs in other organisms. **(A)** Phylogenetic tree of *ser*C1 and *ser*C2 proteins. **(B)** Phylogenetic tree of *tyr*C proteins. Sequences are color-coded according to their genomic location (plastid, plasmid, bacterial chromosome) and the organismal lineage in which they reside. Scale bars indicate the inferred number of amino acid sequences per site.

**Figure 6:**
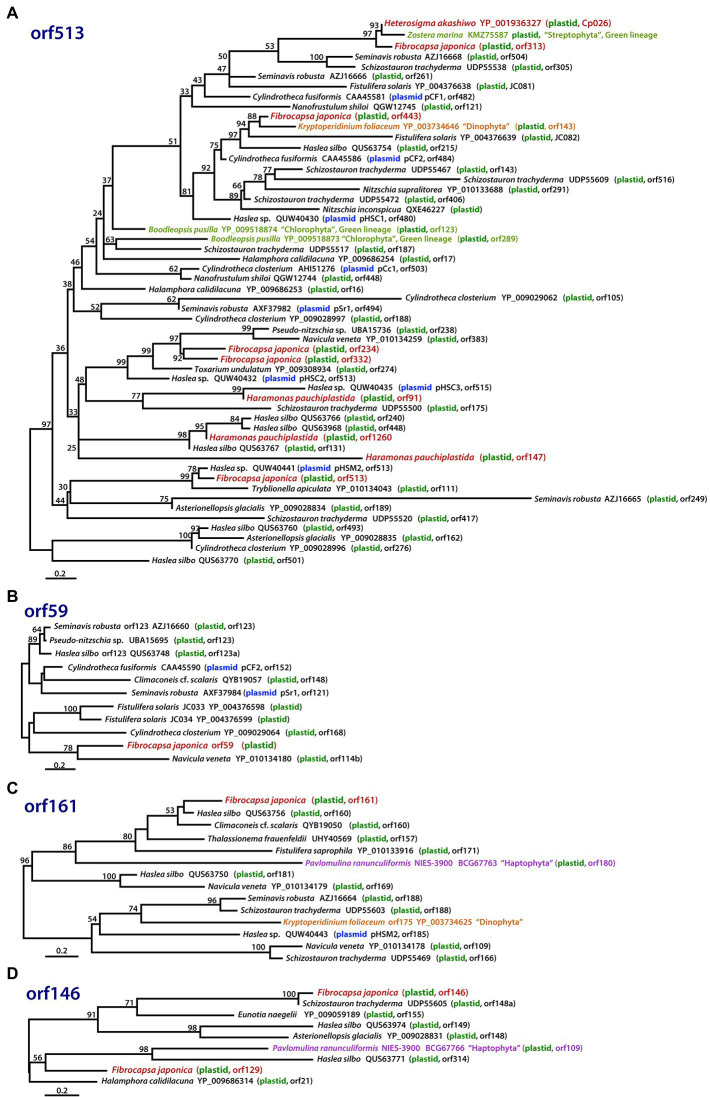
Phylogenies of hypothetical ORFs derived *via* LGT in raphidophytes, diatoms, and other organisms. The phylogenies shown are of proteins with homology to **(A)** ORF513, **(B)** ORF59, **(C)** ORF161, and **(D)** ORF146 in the *Fibrocapsa japonica* plastid genome. Numbers on branches are IQ-Tree UFBoot support values. The scale bar shows the inferred number of amino acid substitutions per site.

Previous work has shown that in red alga-derived complex plastids, most of the lineage-specific genes show complex distribution patterns suggestive of independent losses across a broad range of phylogenetic depths (Kim et al., 2017, 2019). Although the plastid genomes of Raphidophyceae studied herein are generally conserved in structure and gene content, a handful of genes were identified as being lineage-specific (Figures 3, 4A). To better understand the evolutionary distribution and phylogenetic relationships of these patchily distributed genes among eukaryotes, we performed comparative genomic and phylogenetic analyses of plastid homologs from all the major photosynthetic eukaryotic groups relative to their homologs in non-plastid genomes (Figures 4–6). As we shall see, the results support widespread differential loss in primary- and secondary/tertiary plastid-bearing organisms, but also that LGT has resulted in lineage-specific gene gain.

The light-independent protochlorophyllide oxidoreductase (LIPOR) genes involved in the light-independent synthesis of chlorophyll (Shi and Shi, 2006) are present in red-algal plastid genomes. LIPOR arose in anoxygenic photosynthetic bacteria, likely from an ancestral nitrogenase enzyme (Fujita and Bauer, 2003; Muraki et al., 2010). Unlike the gene for POR light-dependent protochlorophyllide oxidoreductase, which has been transferred to the nucleus (or acquired by LGT), LIPOR genes, when present, remain in the plastid (Hunsperger et al., 2015). In lineages with red alga-derived plastids, the three LIPOR genes (*chl*B, *chl*L, and *chl*N) are patchily distributed. They are absent from the plastid genome of the raphidophyte *He. akashiwo* but present in *Chattonella subsalsa* (Hunsperger et al., 2015) and in three of the raphidophycean species examined herein (*V. virescens*, *C. marina*, and *Ha. pauciplastida*; Figure 3), and present as pseudogenes in some but not all sequenced cryptophyte plastid genomes (Fong and Archibald, 2008; Hunsperger et al., 2015; Kim et al., 2017). These results underscore the dynamic evolution of the LIPOR subunit genes in algae with red alga-derived plastids, the functional significance of which is presently unclear (Hunsperger et al., 2015).

The sensor kinase/response regulator protein subunits *tsg*1 and *trg*1 are generally thought to function together in two-component His-to-Asp signal transduction (Duplessis et al., 2007). The presence of this gene pair is linked to a variety of adaptive responses to environmental cues. Although one or both of these genes are found in most rhodophyte plastid genomes, the genes show a variable presence–absence pattern in lineages with red alga-derived secondary/tertiary plastids (Figure 4A). Most plastid genomes of cryptophytes and haptophytes retain both *tsg*1 and *trg*1, while most stramenopile lineages possess neither gene; the raphidophytes are an interesting exception.

Five of the seven raphidophycean plastid genomes analyzed contain the His-to-Asp sensor kinase gene *tsg*1 (transcriptional sensor gene 1); it is absent in the *Ha. pauciplastida* and *F. japonica* plastid genomes. The presence of a plastid-encoded *tsg*1 gene is not universal in red alga-derived plastid-containing lineages (Figure 4A). A plastid *tsg*1 homolog is found in the haptophytes *Emiliania huxleyi, Isochrysis galbana*, *Tisochrysis lutea*, and *Pavlomulina ranunculiformis* (annotated as *dfr*) but missing in *Chrysochromulina*, *Diacronema*, and *Phaeocystis*. In cryptophytes, the *tsg*1 gene is present in five species but missing in the plastid genomes of *Cryptomonas* spp. and *Guillardia theta*. In stramenopiles, *tsg*1 is only found in raphidophyceans; its absence is notable in the bulk of sequenced plastid-bearing members (bacillariophytes, Bolidophyceae, Chrysophyceae, Dictyochophyceae, Eustigmatophyceae, Olisthodiscophyceae, Pelagophyceae, Phaeophyceae, Xanthophyceae). In rhodophytes, the *tsg*1 gene (annotated as *ycf*26 or *dfr*) is encoded in the plastid genomes of Bangiophyceae, Compsopogonophyceae, Stylonematophyceae, most florideophycean species, and some Cyanidiophyceae (*Cyanidium caldarium*, *Cyanidiococcus yangmingshanensis*, as a pseudogene in *Galdieria sulphuraria*, missing in *Cyanidioschyzon merolae*), but missing in other rhodophycean algal groups (e.g., Porphyridiophyceae and Rhodellophyceae). In glaucophytes, the gene is present in *Glaucocystis* spp. but missing in studied *Cyanophora* species. The *tsg*1 gene has also been identified in the “chromatophore” genome of the rhizarian testate amoeba *Paulinella* (the chromatophore is a photosynthetic organelle that evolved independent of canonical plastids; Macorano and Nowack, 2021).

Together with *tsg*1, *trg*1 (transcriptional response regulator gene 1, annotated as *trg*1, *ycf*27, *orf*27, or *omp*R) was found in five raphidophycean species, but missing in *Ha. pauciplastida* and *F. japonica*, which also lack *tsg*1 (Figure 4A). Beyond the raphidophytes, the distribution of *trg*1 is varied among disparate taxa, but generally present more often than is the *tsg*1 gene in red alga-derived secondary/tertiary plastids. As with *tsg*1, the *trg*1 gene is missing in other stramenopiles. The raphidophycean *tsg*1 gene/protein appears more closely related to homologs in Cyanidiophyceae and Stylonematophyceae (Rhodophyta) than to Cryptophyta and Haptophyta, although the phylogenetic tree topology is not robust in this regard (Figure 4B; Supplementary Figure S3).

The presence–absence patterns of *tsg*1 and *trg*1 in sequenced plastid genomes is complex. The presumptive loss of the sensor kinase in some plastid genomes suggests that under such circumstances, the regulatory protein may be governed by one or more nuclear-encoded sensor kinases or by as-yet undescribed accessory proteins that are either of nuclear or plastid origin. Given that most of the raphidophyte plastid genomes sequenced herein encode a single response regulator and its cognate sensor kinase, but other stramenopile plastid genomes lack both genes, the extent to which nuclear genes participate in this signal transduction system in stramenopiles is unclear.

### Lineage-Specific Gene Gain by LGT

The *ser*C gene encodes a phosphoserine aminotransferase, which in algae has thus far only been found in red alga-derived secondary plastid genomes, specifically those of diatoms (Figure 5A; Brembu et al., 2014; Ruck et al., 2014; Hamsher et al., 2019; Li et al., 2019; Gastineau et al., 2021). Interestingly, the diatom plastid *ser*C has strong similarity to plasmid DNAs of several diatoms (e.g., *Cylindrotheca fusiformis* and *Haslea* sp.; Figure 5A, blue) and the diatom-derived plastid genome of the “dinotom” *Kryptoperidinium foliaceum* (Figure 5A, orange-brown). Unexpectedly, we found a *ser*C homolog in the plastid genome of the marine raphidophyte *F. japonica* that in a protein phylogeny branches specifically with the plasmid-borne sequences of *C. fusiformis* and *C. closterium* and a plastid homolog in *Climaconeis* cf. *scalaris*. *Fibrocapsa japonica* also has a “*ser*C2-like” gene with very high similarity to the plastid *ser*C2 of the diatom *Plagiogrammopsis vanheurckii* and one of four *ser*C homologs in *Schizostauron trachyderma* (Figure 5A). One explanation for the presence of these genes in *F. japonica* is that they were acquired recently by LGT from a diatom plastid and/or plasmid DNA, perhaps by mixotrophic feeding. This scenario may also explain the existence of the *ser*C homolog in *K. foliaceum*. From where exactly the plasmid and plastid *ser*C homologs of diatoms originated is unclear; these sequences are embedded within the plastid and plasmid clade, very distinct from the bacterial homologs (*e*-value cutoff = 1e^−5^, word size = 6) used to root the *ser*C1 / *ser*C2 tree (Figure 5A; Supplementary Figure S4).

The *tyr*C gene encodes a putative site-specific tyrosine recombinase in the *He. akashiwo* and *F. japonica* plastid genomes (Figures 3, 5B). The *tyr*C gene has also been found in other diatoms, “dinotoms,” green algae, and select bacteria; in phylogenetic analyses, the raphidophyte homologs are more similar to sequences in pennate diatoms than to green algal homologs (Figure 5B). As is the case for *ser*C, the presence of *tyr*C in the *He. akashiwo* and *F. japonica* genomes (as well as a *tyr*C pseudogene in *F. japonica*) is consistent with LGT from a diatom, though it is worth noting that the *He. akashiwo* and *F. japonica* sequences are not monophyletic. In our phylogenies, two *He. akashiwo tyr*C homologs are nested deeply within a strongly supported clade of diatom sequences (and the “dinotom” of *K. foliaceum*), far away from two *F. japonica* sequences, which are themselves not monophyletic; some or all of these sequences may have been acquired independent of one another. *Ulva fasciata* (Chlorophyta), *Netrium digitus* (Streptophyta), and various other green algae possess *tyr*C genes in their plastid genomes (Figure 5B; Brouard et al., 2008; Civáň et al., 2014). In bacteria, *xer*C/D family tyrosine recombinases with a lower degree of sequence similarity (*e-*value cutoff = 1e^−5^, word size = 6) to *tyr*C are found primarily in Firmicutes. The specific evolutionary connections between the raphidophyte, diatom, and green algal *tyr*C homologs to one another and to bacterial *xer*C/D are unclear.

Further evidence for the role of LGT in the evolution of raphidophyte plastid genomes comes from our discovery of a group II intron in the *psb*B gene of *F. japonica*, a feature not described in raphidophytes thus far (Figure 3). Interestingly, the intron contains an intron-encoded protein (IEP) with a reverse transcriptase (RT) domain that is clearly related to IEPs in other red alga-derived plastid genomes, such as those in the group II introns of the *dna*K, *psa*A and *trn*M genes of rhodophytes, *ch*lB, *psa*A, and *psb*B in cryptophytes, in the *psa*J gene of the green alga-derived plastid genome of euglenoids, as well as various plastid and/or mitochondrial genes in other stramenopiles, fungi, and rhizarians (Supplementary Figure S5). This is consistent with earlier studies documenting the patchy distribution of group II introns in cyanobacteria and diverse eukaryotes (e.g., Odom et al., 2004; Sheveleva and Hallick, 2004; Khan and Archibald, 2008; Kim et al., 2022; Suzuki et al., 2022). While our phylogenetic analyses failed to identify an obvious donor of the *F. japonica psb*B intron, it is noteworthy that previous investigation of the mitochondrial genome of the raphidophyte *Chattonella marina* identified a putative LGT of a group II intron from diatoms to raphidophytes (Kamikawa et al., 2009). More specifically, phylogenetic analysis of the RT domain of the mitochondrial *cox*1 IEP in *C. marina* showed that it clustered with *cox*1 IEPs of the diatom *Thalassiosira pseudonana* and the pennate diatom obligate endosymbiont in the dinoflagellate *Kryptoperidinium foliaceum*. We also discovered a 2,472 nt intron found only in the *clp*C gene of the marine raphidophycean species *Ha. pauciplastida*, but its origin is unknown as it does not contain an obvious and analyzable IEP (the intron nucleotide sequence shows no obvious significant similarity to known introns). Detailed speculation on the frequency and directionality of group II intron transfers in eukaryotes is beyond the scope of our study. But we should emphasize that mitochondrial IEPs, such as those analyzed by Kamikawa et al. (2009) and Kim et al. (2022), were only rarely retrieved in our BLAST sequence searches (*e-*value cutoff = 1e^−5^, word size = 6) and are thus poorly represented in the RT / IEP phylogenetic analyses presented herein (the *F. japonica* plastid *psb*B intron and the *C. marina* mitochondrial *cox*1 IEP are too distant from one another to allow for meaningful comparison). All these caveats aside, our results are consistent in showing intriguing connections between mobile genetic elements in diatoms and marine raphidophytes.

### Additional “Foreign” Sequences in the *Fibrocapsa japonica* Plastid Genome

Examples of LGT-derived plastid genes and introns in algae are increasingly well known, such as in rhodophytes (Janouškovec et al., 2010), cryptophytes (Khan and Archibald, 2008; Kim et al., 2017; Suzuki et al., 2022), haptophytes (Rice and Palmer, 2006), chrysophytes (Kim et al., 2019), diatoms (Ruck et al., 2014), eustigmatophytes (Yurchenko et al., 2016), and the raphidophyte *He. akashiwo* (Cattolico et al., 2008). Curiously, some of these foreign sequences appear to have been acquired from plasmid DNAs, portions of which are found inserted into the plastid genomes of several diatoms and encode putatively functional site-specific recombinase genes (Brembu et al., 2014; Ruck et al., 2014; Gastineau et al., 2021). They are also found in “dinotoms” (Imanian et al., 2010), raphidophytes (Cattolico et al., 2008), and red algae (Lee et al., 2016). Further investigation is required to determine if the plastid-encoded DNA recombinases in disparate algal lineages are actually involved in site-specific recombination and integration of foreign sequences.

The six gene-poor regions in the *F. japonica* plastid genome sequenced in our study were found to contain several hypothetical ORFs derived from various pennate diatom plastids or plasmid DNAs (Figure 2). In fact, hypothetical ORFs with similarity (*e-*value cutoff = 1e^−5^, word size = 6) to ORFs in the plasmid DNAs of the diatoms *Cylindrotheca closterium*, *Cylindrotheca fusiformis*, *Seminavis robusta,* and *Haslea* species are found not just in the plastid genome of *F. japonica*, but *He. akashiwo* and *Ha. pauciplastida* as well (Figures 6A–C). These sequences are also homologous to predicted genes in the plastids of some chlorophyte green algae (Figure 6A), the “dinotom” *K. foliaceum* (Figures 6A,C), and haptophytes (Figures 6C,D). Two hypothetical ORFs of *F. japonica* share homology with pennate diatom plastid sequences: orf136 is homologous to *Seminavis robusta* orf140 (*e*-value = 8.96*e*^−25^) and *Toxarium undulatum* orf127 (*e*-value = 6.92*e*^−26^), and orf153 is homologous to *Asterionellopsis glacialis* orf126 (*e*-value = 1.95*e*^−02^). How the plastid genomes of these disparate algae have come to contain these sequences is unclear, but it seems likely that plasmid-mediated gene exchange was somehow involved.

### The Evolution of Raphidophytes and Their Mosaic Plastid Genomes

**Figure 7:**
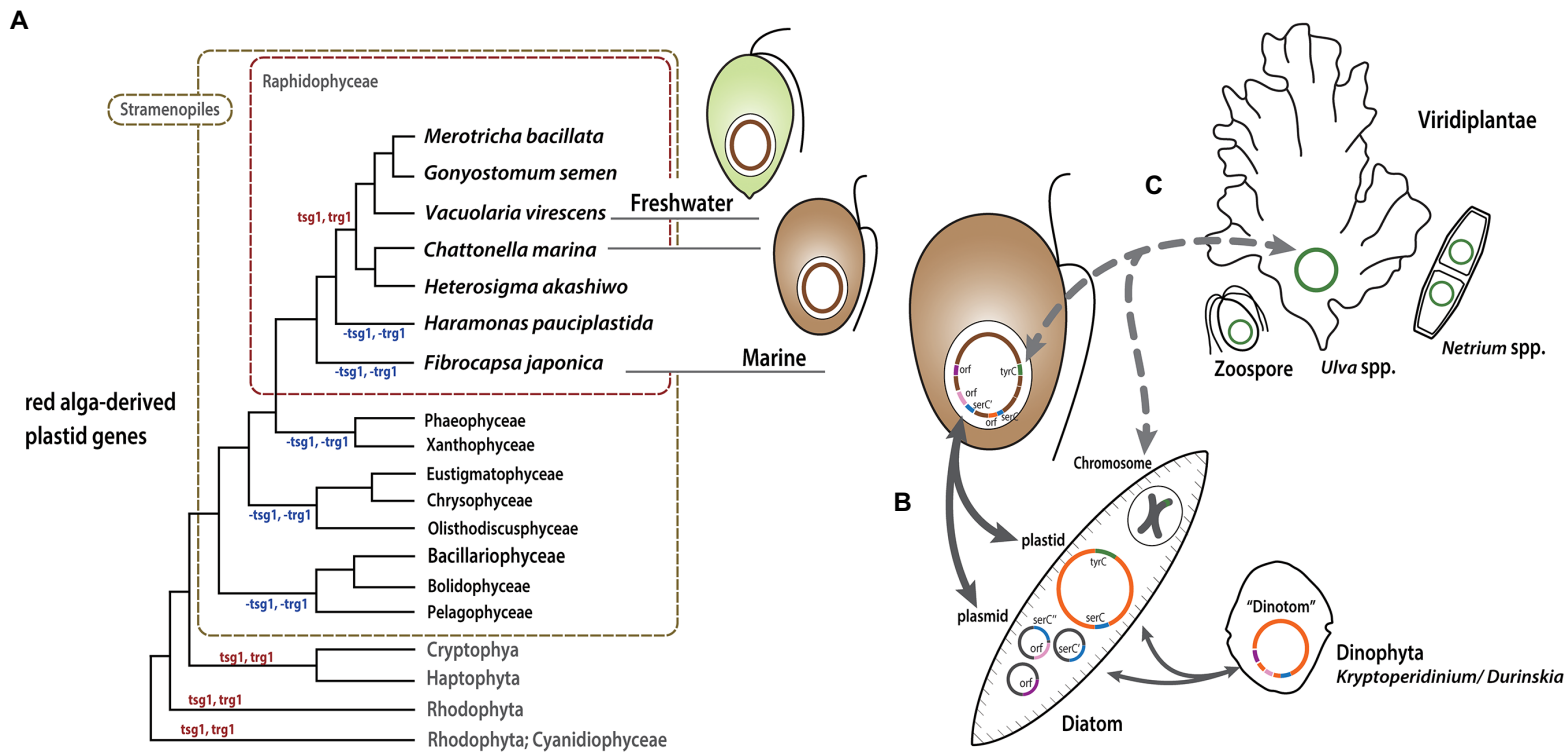
Model of Raphidophyceae plastid evolution. Putative gene gain/loss and LGT events are mapped onto a schematic tree **(A)** modified from the phylogeny inferred from plastid proteins shown in Supplementary Figure S1. **(B)** and **(C)** show possible routes of gene exchange between the organisms described in the text.

Raphidophyceae are mixotrophs that photosynthesize as well as feed on diverse bacteria and organic nutrients in freshwater and marine environments. The marine raphidophytes *Chattonella* spp., *He. akashiwo*, and *Fibrocapsa* spp. feed on heterotrophic and autotrophic bacteria by capturing prey cells/microspheres in mucus excreted by mucocysts (Jeong, 2011). We have shown that the freshwater raphidophycean taxa have small plastid genomes relative to those of marine species, and the comparative genomic and phylogenetic analyses carried out here and elsewhere (Cattolico et al., 2008) suggest a link between mixotrophy, plastid genome expansion, and LGT. How and how often LGT has occurred is still unclear. Nevertheless, based on our results we propose a model of raphidophycean plastid evolution (Figure 7). The red alga-type plastid of Raphidophyceae stems from a secondary or possibly tertiary endosymbiotic event in an ancestor shared with other plastid-bearing stramenopiles (Figure 7A; Supplementary Figure S1; Kim et al., 2017, 2019, 2020; Sibbald and Archibald, 2020). The presence and distribution of *tsg*1 and *trg*1 genes is consistent with tertiary endosymbiotic linkages between raphidophytes, haptophytes, and cryptophytes, although the most likely donor(s) and recipients are not clear from these data alone (Figure 4A). After the diversification of Raphidophyceae, some mixotrophic marine species appear to have taken up mobile elements in plastid and/or plasmid DNAs by feeding on eukaryotes, such as pennate diatoms, with genes being transferred to their plastid genomes (Figure 7B). Plastid genome expansion and gene rearrangement thus occurred as a result of—and/or was facilitated by—acquisition of foreign genetic material by LGT. This pattern has been detected in the “dinotom” *Kryptoperidinium* and *Durinskia* species (Imanian et al., 2010). As noted above, the evolution of the raphidophyte LGT-derived *tyr*C genes in *He. akashiwo* and *F. japonica* is unclear. Some of these genes could conceivably could have been acquired directly by feeding on tiny green algal cells, or indirectly from other sources (e.g., diatoms; Figure 7C). Together with LGT-associated plastid genome expansion in marine mixotrophic species, contraction of the IR region in the plastid genome of the freshwater species *G. semen* represents an example of genome reduction “in action,” which is also consistent with our model.

## Conclusion

Analysis of six newly sequenced plastid genomes from Raphidophyceae has provided insight into organellar genome dynamics. The raphidophycean lineages evolved from a common ancestor shared with diatoms and other red alga plastid-bearing stramenopiles, with LGT having increased the coding capacity of mixotrophic species on multiple occasions during raphidophycean algal evolution. While the extent to which LGT has contributed to the plastid genomes of Raphidophyceae and other algae remains to be seen, our results indicate that plasmids may play an important role. Our understanding of the diversity and biology of such mobile elements in eukaryotes is still very limited, and the discovery and characterization of plasmids in diverse algae will hopefully provide the data with which to test this hypothesis.

## Data Availability Statement

The datasets presented in this study can be found in GenBank under the following accession numbers: ON228255–ON228260.

## Author Contributions

JIK and WS conceived and designed the experiments. JIK, BYJ, MGP, and YDY isolated cells and provided cultures. JIK performed the experiments and analyzed the data. JIK, WS, and JMA interpreted the data and wrote the manuscript. All authors contributed to the article and approved the submitted version.

## Funding

This study was supported by funds from the National Research Foundation (NRF) of Korea awarded to JIK (NRF-2018R1D1A1B07050727 and 2021R1C1C2012996), BYJ (NRF-2014R1A1A3052827), MGP (NRF-2016R1A6A1A03012647), YDY (NRF-2021M 3I6A1091272), and WS (NRF-2019R1I1A2A01063159), and a Natural Sciences and Engineering Research Council of Canada grant (RGPIN-2019-05058) awarded to JMA.

## Conflict of Interest

The authors declare that the research was conducted in the absence of any commercial or financial relationships that could be construed as a potential conflict of interest.

## Publisher’s Note

All claims expressed in this article are solely those of the authors and do not necessarily represent those of their affiliated organizations, or those of the publisher, the editors and the reviewers. Any product that may be evaluated in this article, or claim that may be made by its manufacturer, is not guaranteed or endorsed by the publisher.

## Footnotes

^1^ http://bioinf.spbau.ru/spades

^2^ http://exon.gatech.edu/genemark/genemarks.cgi

^3^ http://www.geneious.com/

^4^ http://lowelab.ucsc.edu/tRNAscan-SE/

^5^ http://megasun.bch.umontreal.ca/cgi-bin/RNAweasel/RNAweaselInterface.pl

^6^ http://ogdraw.mpimp-golm.mpg.de/

^7^ http://tree.bio.ed.ac.uk/software/figtree/

## Supplementary Material

Supplementary Figure S1 Phylogenetic tree of raphidophyte plastids. This tree was constructed using a dataset of 91 plastid genes (18,216 amino acids) from 135 taxa. The numbers on each node represent ultrafast bootstrap approximation (UFBoot) values calculated using IQ-Tree. The scale bar indicates the number of amino acid substitutions per site.

Supplementary Figure S2 Circular map of newly sequenced plastid genomes of Raphidophyceae. The lineage-specific gene gains are labeled inside or outside of the circles.

Supplementary Figure S3 **(A)** Expanded phylogenetic tree for Figure 4B based on tsg1 amino acid sequences. The tree was constructed using a dataset assembled by BLASTp (e-value cutoff = 1e^−5^) against the NCBI non-redundant database using the raphidophyte homologs as queries. **(B)** Protein alignment of homologs used for phylogenetic reconstruction.

Supplementary Figure S4 **(A)** Expanded phylogenetic tree for Figure 5B based on tyrC amino acid sequences from *F. japonica* and detectable homologs in other organisms. The dataset was assembled using sequences retrieved by BLASTp (e-value cutoff = 1e^−5^) against the NCBI non-redundant database using raphidophyte homologs as queries. **(B,C)** Aligned amino acid sequences of serC and tyrC, respectively.

Supplementary Figure S5 Phylogenetic tree based on the reverse transcriptase homologs of the intron-encoded protein in the *psb*B group II intron of *F. japonica*. The location of the intron is shown after the gene names [e.g., rnl-i1 = intron 1 in rnl or *psb*B (with single intron IEP)]. The tree was constructed using a dataset of homologs retreived by BLASTp (e-value cutoff = 1e^−5^) from the NCBI non-redundant database using the *F. japonica* *psb*B group II intron IEP as a query. Numbers (Bootstrap values > 50%) on branches are IQ-Tree UFBoot. The scale bar shows the inferred number of amino acid substitutions per site.
