# Supplementary figures and images for: Evolutionary Dynamics and Lateral Gene Transfer in Raphidophyceae Plastid Genomes
Source: Front Plant Sci. 2022 May 26;13:896138. doi: 10.3389/fpls.2022.896138 (PMC9235467; doi:10.3389/fpls.2022.896138)

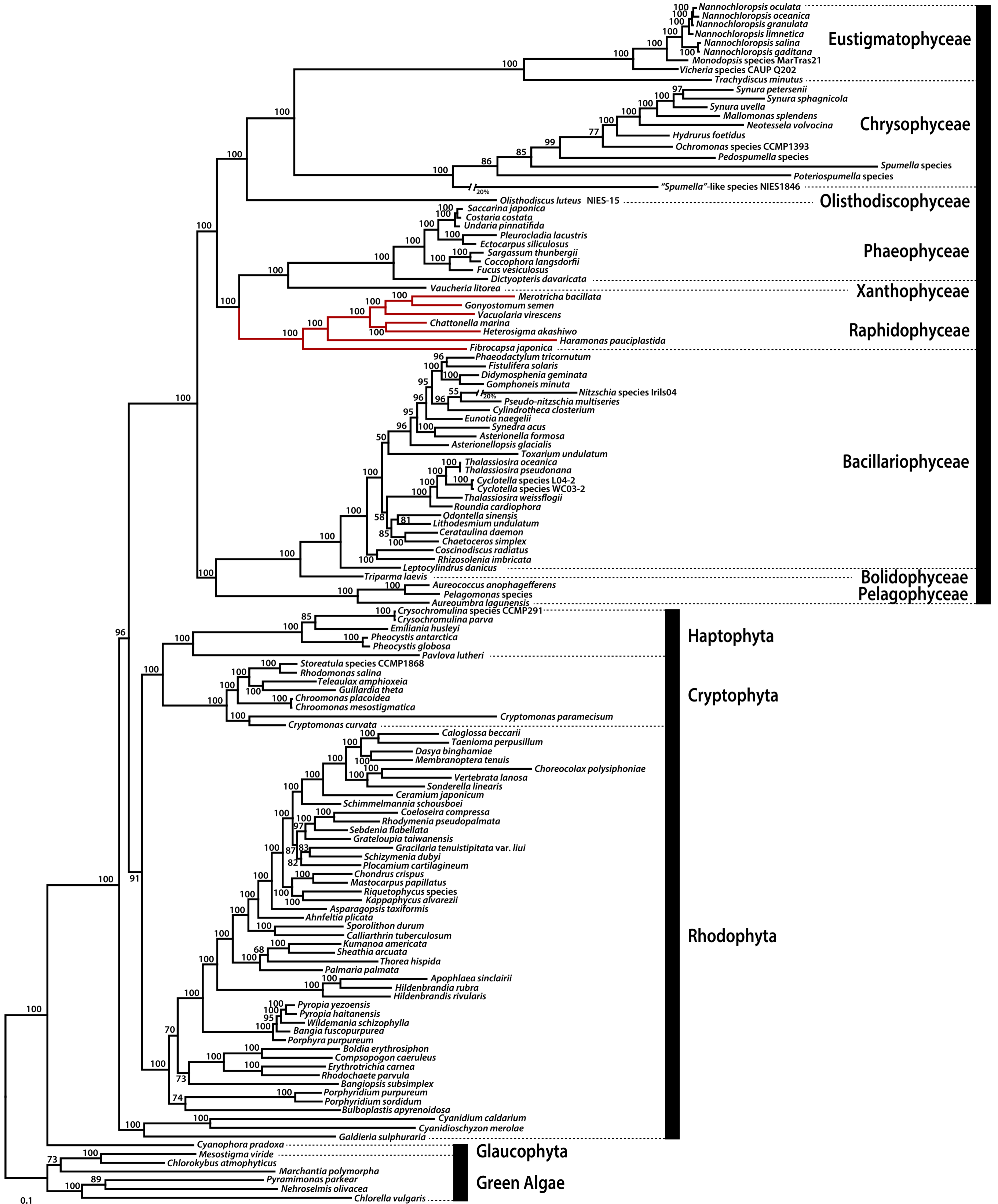

Supplement: Supplementary Figure S1 — Phylogenetic tree of raphidophyte plastids. This tree was constructed using a dataset of 91 plastid genes (18,216 amino acids) from 135 taxa. The numbers on each node represent ultrafast bootstrap approximation (UFBoot) values calculated using IQ-Tree. The scale bar indicates the number of amino acid substitutions per site. [file Data_Sheet_1.PDF]
