# Supplementary material for: Evolutionary Dynamics and Lateral Gene Transfer in Raphidophyceae Plastid Genomes
Source: Front Plant Sci. 2022 May 26;13:896138. doi: 10.3389/fpls.2022.896138 (PMC9235467; doi:10.3389/fpls.2022.896138)
Supplement: Supplementary Figure S3 — (A) Expanded phylogenetic tree for Figure 4B based on tsg1 amino acid sequences. The tree was constructed using a dataset assembled by BLASTp (e-value cutoff = 1e−5) against the NCBI non-redundant database using the raphidophyte homologs as queries. (B) Protein alignment of homologs used for phylogenetic reconstruction. [file Data_Sheet_3.PDF]

A. tree of *tsg*1

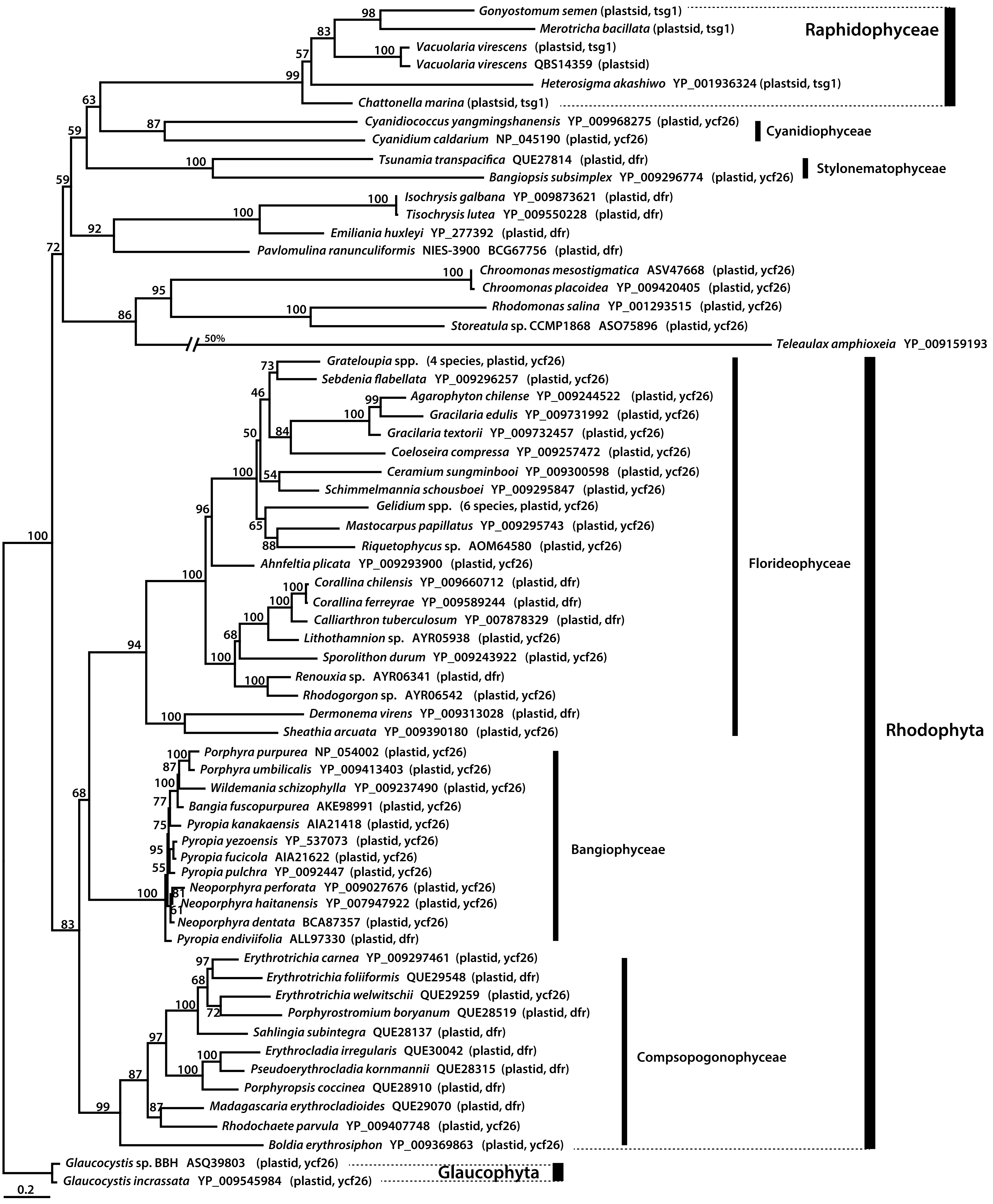

B. alignment of *tsg*1

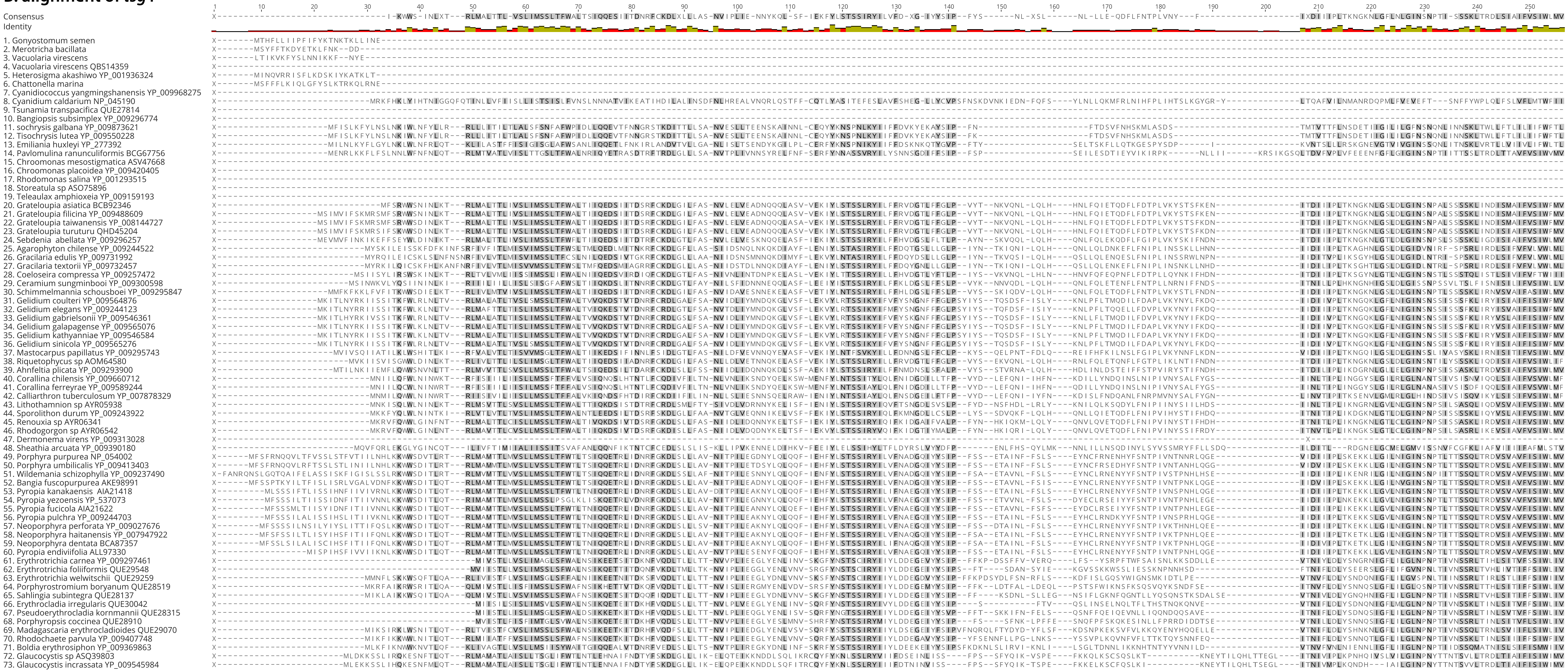

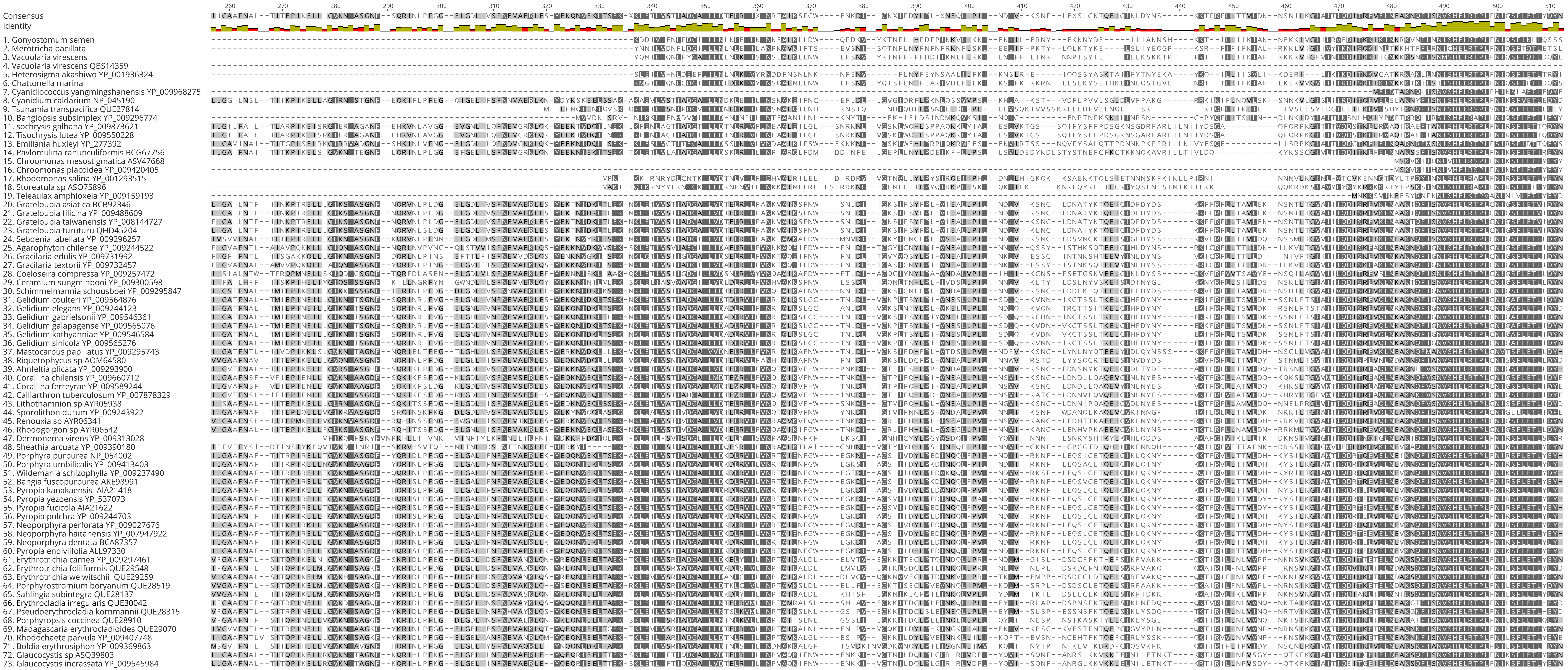
260
270
280
290
300
310
320
330
340
350
360
370
380
390
400
410
420
430
440
450
460
470
480
490
500
510
Consensus
Identity
1. Gonyostomum semen
2. Merotricha bacillata
3. Vacuolaria virescens
4. Vacuolaria virescens QBS14359
5. Heterosigma akashiwo YP_001936324
6. Chattonella marina
7. Cyanidiococcus yangmingshanensis YP_009968275
8. Cyanidium caldarium NP_045190
9. Tsunamia transpacifica QUE27814
10. Bangiopsis subsimplex YP_009296774
11. sochrysis galbana YP_009873621
12. Tisochrysis lutea YP_009550228
13. Emiliania huxleyi YP_277392
14. Pavlomulina ranunculiformis BCG67756
15. Chroomonas mesostigmatica ASV47668
16. Chroomonas placoidea YP_009420405
17. Rhodomonas salina YP_001293515
18. Storeatula sp ASO75896
19. Teleaulax amphioxeia YP_009159193
20. Grateloupia asiatica BCB92346
21. Grateloupia filicina YP_009488609
22. Grateloupia taiwanensis YP_008144727
23. Grateloupia turuturu QHD45204
24. Sebdenia abellata YP_009296257
25. Agarophyton chilense YP_009244522
26. Gracilaria edulis YP_009731992
27. Gracilaria textorii YP_009732457
28. Coeloseira compressa YP_009257472
29. Ceramium sungminbooi YP_009300598
30. Schimmelmannia schousboei YP_009295847
31. Gelidium coulteri YP_009564876
32. Gelidium elegans YP_009244123
33. Gelidium gabrielsonii YP_009546361
34. Gelidium galapagense YP_009565076
35. Gelidium kathyanniae YP_009546584
36. Gelidium sinicola YP_009565276
37. Mastocarpus papillatus YP_009295743
38. Riquetophycus sp AOM64580
39. Ahnfeltia plicata YP_009293900
40. Corallina chilensis YP_009660712
41. Corallina ferreyrae YP_009589244
42. Calliarthron tuberculosum YP_007878329
43. Lithothamnion sp AYR05938
44. Sporolithon durum YP_009243922
45. Renouxia sp AYR06341
46. Rhodogorgon sp AYR06542
47. Dermonema virens YP_009313028
48. Sheathia arcuata YP_009390180
49. Porphyra purpurea NP_054002
50. Porphyra umbilicalis YP_009413403
51. Wildemania schizophylla YP_009237490
52. Bangia fuscopurpurea AKE98991
53. Pyropia kanakaensis AIA21418
54. Pyropia yezoensis YP_537073
55. Pyropia fucicola AIA21622
56. Pyropia pulchra YP_009244703
57. Neoporphyra perforata YP_009027676
58. Neoporphyra haitanensis YP_007947922
59. Neoporphyra dentata BCA87357
60. Pyropia endiviifolia ALL97330
61. Erythrotrichia carnea YP_009297461
62. Erythrotrichia foliiformis QUE29548
63. Erythrotrichia welwitschii QUE29259
64. Porphyrostromium boryanum QUE28519
65. Sahlingia subintegra QUE28137
66. Erythrocladia irregularis QUE30042
67. Pseudoerythrocladia kornmannii QUE28315
68. Porphyropsis coccinea QUE28910
69. Madagascaria erythrocladioides QUE29070
70. Rhodochaete parvula YP_009407748
71. Boldia erythrosiphon YP_009369863
72. Glaucocystis sp ASQ39803
73. Glaucocystis incrassata YP_009545984

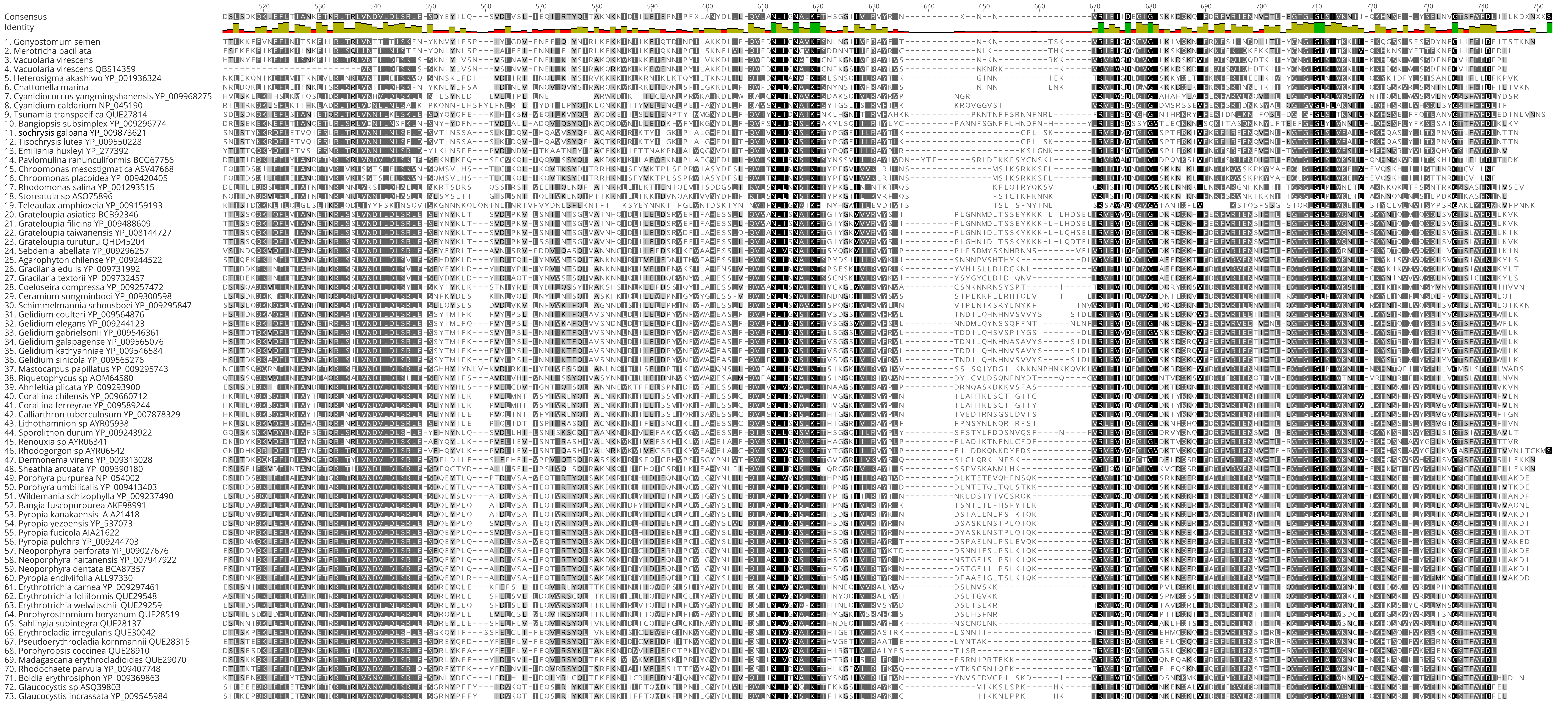
520
530
540
550
560
570
580
590
600
610
620
630
640
650
660
670
680
690
700
710
720
730
740
750
Consensus
Identity
1. Gonyostomum semen
2. Merotricha bacillata
3. Vacuolaria virescens
4. Vacuolaria virescens QBS14359
5. Heterosigma akashiwo YP_001936324
6. Chattonella marina
7. Cyanidiococcus yangmingshanensis YP_009968275
8. Cyanidium caldarium NP_045190
9. Tsunamia transpacifica QUE27814
10. Bangiopsis subsimplex YP_009296774
11. sochrysis galbana YP_009873621
12. Tisochrysis lutea YP_009550228
13. Emiliania huxleyi YP_277392
14. Pavlomulina ranunculiformis BCG67756
15. Chroomonas mesostigmatica ASV47668
16. Chroomonas placoidea YP_009420405
17. Rhodomonas salina YP_001293515
18. Storeatula sp ASO75896
19. Teleaulax amphioxeia YP_009159193
20. Grateloupia asiatica BCB92346
21. Grateloupia filicina YP_009488609
22. Grateloupia taiwanensis YP_008144727
23. Grateloupia turuturu QHD45204
24. Sebdenia abellata YP_009296257
25. Agarophyton chilense YP_009244522
26. Gracilaria edulis YP_009731992
27. Gracilaria textorii YP_009732457
28. Coeloseira compressa YP_009257472
29. Ceramium sungminbooi YP_009300598
30. Schimmelmannia schousboei YP_009295847
31. Gelidium coulteri YP_009564876
32. Gelidium elegans YP_009244123
33. Gelidium gabrielsonii YP_009546361
34. Gelidium galapagense YP_009565076
35. Gelidium kathyanniae YP_009546584
36. Gelidium sinicola YP_009565276
37. Mastocarpus papillatus YP_009295743
38. Riquetophycus sp AOM64580
39. Ahnfeltia plicata YP_009293900
40. Corallina chilensis YP_009660712
41. Corallina ferreyrae YP_009589244
42. Calliarthron tuberculosum YP_007878329
43. Lithothamnion sp AYR05938
44. Sporolithon durum YP_009243922
45. Renouxia sp AYR06341
46. Rhodogorgon sp AYR06542
47. Dermonema virens YP_009313028
48. Sheathia arcuata YP_009390180
49. Porphyra purpurea NP_054002
50. Porphyra umbilicalis YP_009413403
51. Wildemania schizophylla YP_009237490
52. Bangia fuscopurpurea AKE98991
53. Pyropia kanakaensis AIA21418
54. Pyropia yezoensis YP_537073
55. Pyropia fucicola AIA21622
56. Pyropia pulchra YP_009244703
57. Neoporphyra perforata YP_009027676
58. Neoporphyra haitanensis YP_007947922
59. Neoporphyra dentata BCA87357
60. Pyropia endiviifolia ALL97330
61. Erythrotrichia carnea YP_009297461
62. Erythrotrichia foliiformis QUE29548
63. Erythrotrichia welwitschii QUE29259
64. Porphyrostromium boryanum QUE28519
65. Sahlingia subintegra QUE28137
66. Erythrocladia irregularis QUE30042
67. Pseudoerythrocladia kornmannii QUE28315
68. Porphyropsis coccinea QUE28910
69. Madagascaria erythrocladioides QUE29070
70. Rhodochaete parvula YP_009407748
71. Boldia erythrosiphon YP_009369863
72. Glaucocystis sp ASQ39803
73. Glaucocystis incrassata YP_009545984
