# Supplementary material for: Evolutionary Dynamics and Lateral Gene Transfer in Raphidophyceae Plastid Genomes
Source: Front Plant Sci. 2022 May 26;13:896138. doi: 10.3389/fpls.2022.896138 (PMC9235467; doi:10.3389/fpls.2022.896138)

A. tree of *ser*C1/*ser*C2

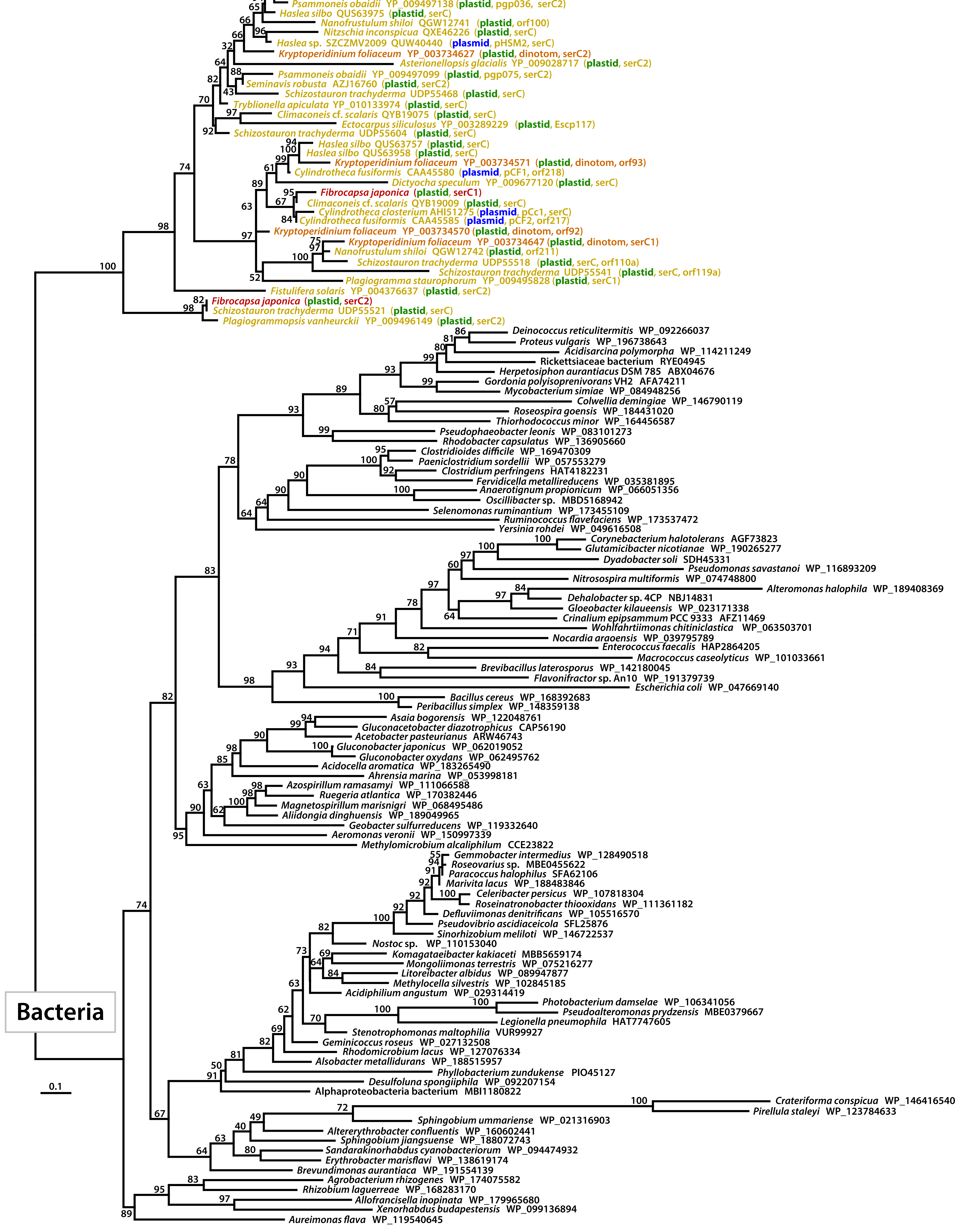

B. alignment of *ser*C1/*ser*C2

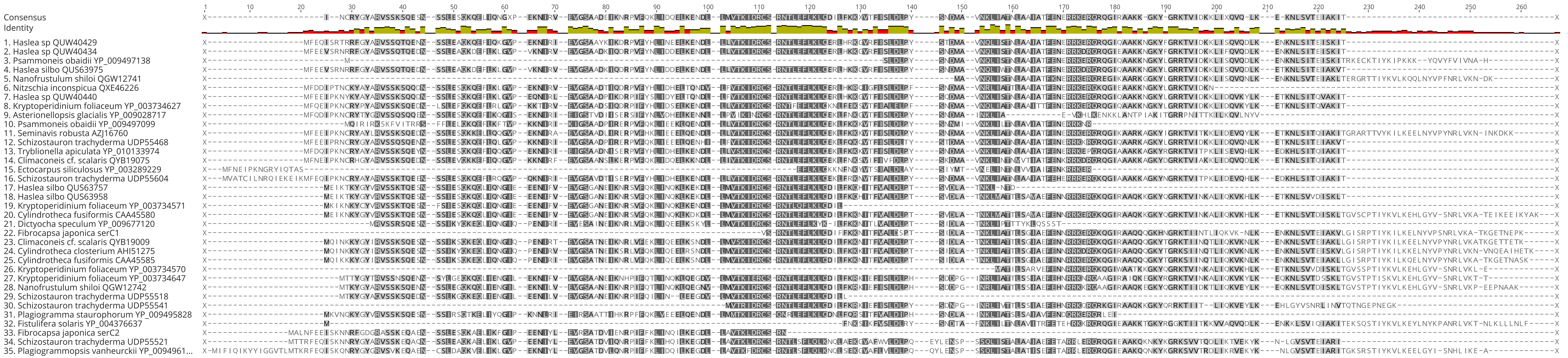

C. alignment of *tyr*C

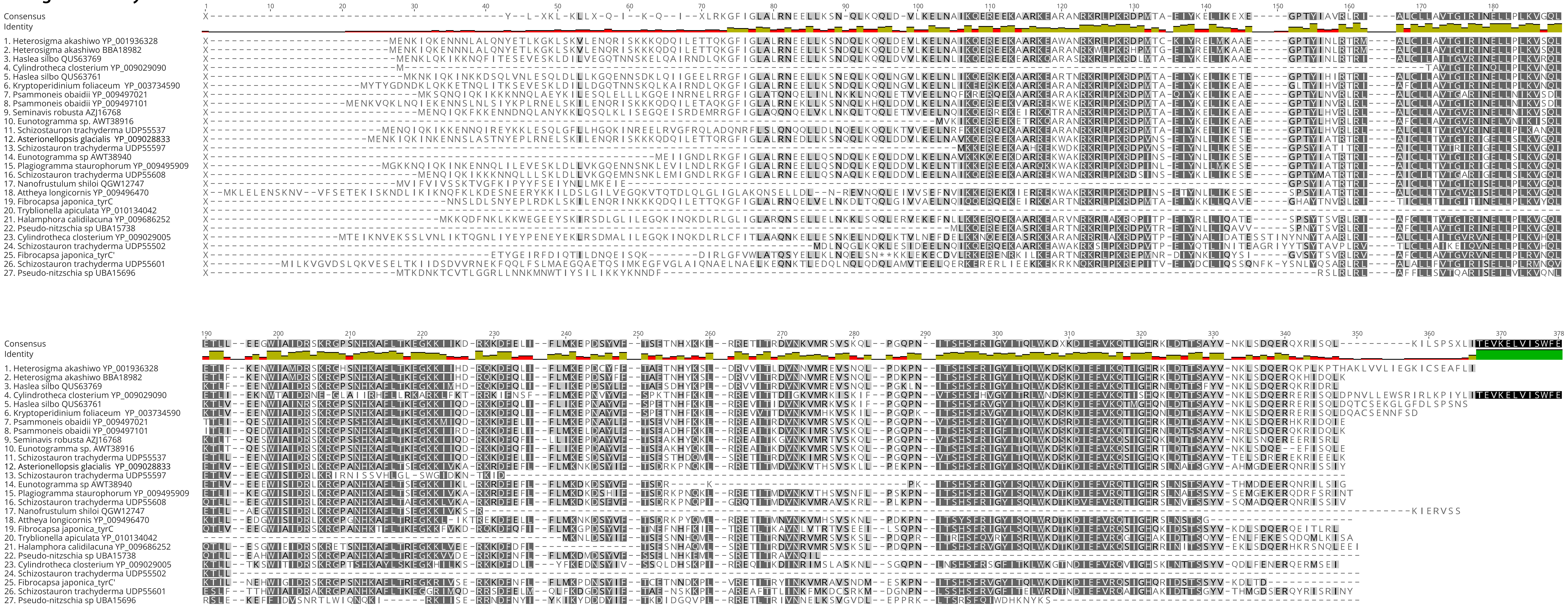

Supplement: Supplementary Figure S4 — (A) Expanded phylogenetic tree for Figure 5B based on tyrC amino acid sequences from F. japonica and detectable homologs in other organisms. The dataset was assembled using sequences retrieved by BLASTp (e-value cutoff = 1e−5) against the NCBI non-redundant database using raphidophyte homologs as queries. (B,C) Aligned amino acid sequences of serC and tyrC, respectively. [file Data_Sheet_4.PDF]
