# Supplementary material for: Evolutionary Dynamics and Lateral Gene Transfer in Raphidophyceae Plastid Genomes
Source: Front Plant Sci. 2022 May 26;13:896138. doi: 10.3389/fpls.2022.896138 (PMC9235467; doi:10.3389/fpls.2022.896138)
Supplement: Supplementary Figure S2 — Circular map of newly sequenced plastid genomes of Raphidophyceae. The lineage-specific gene gains are labeled inside or outside of the circles. [file Data_Sheet_2.PDF]

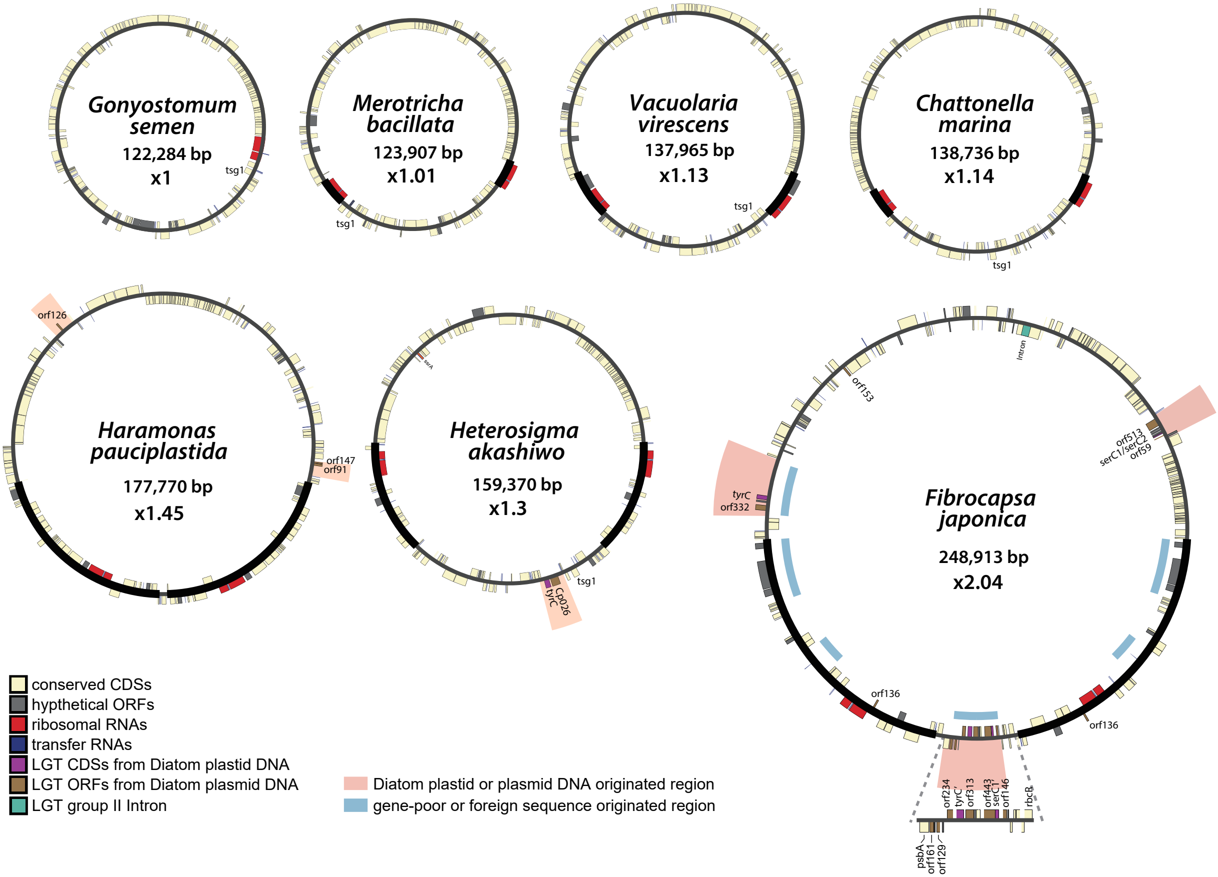

Gonyostomum semen
122,284 bp
x1
tsg1
Merotricha bacillata
123,907 bp
x1.01
tsg1
Vacuolaria virescens
137,965 bp
x1.13
tsg1
Chattonella marina
138,736 bp
x1.14
tsg1
Haramonas pauciplastida
177,770 bp
x1.45
orf126
orf147
orf91
Heterosigma akashiwo
159,370 bp
x1.3
tsg1
Cp026
tyrC
Fibrocapsa japonica
248,913 bp
x2.04
Intron
orf153
orf513
serC1/serC2
orf59
tyrC
orf332
orf136
orf136
orf234
tyrC'
orf313
orf443
serC1'
orf146
rbcR
psbA
orf161
orf129
conserved CDSs
hypthetical ORFs
ribosomal RNAs
transfer RNAs
LGT CDSs from Diatom plastid DNA
LGT ORFs from Diatom plasmid DNA
LGT group II Intron
Diatom plastid or plasmid DNA originated region
gene-poor or foreign sequence originated region
