# Supplementary material for: Evolutionary Dynamics and Lateral Gene Transfer in Raphidophyceae Plastid Genomes
Source: Front Plant Sci. 2022 May 26;13:896138. doi: 10.3389/fpls.2022.896138 (PMC9235467; doi:10.3389/fpls.2022.896138)
Supplement: Supplementary Figure S5 — Phylogenetic tree based on the reverse transcriptase homologs of the intron-encoded protein in the psbB group II intron of F. japonica. The location of the intron is shown after the gene names [e.g., rnl-i1 = intron 1 in rnl or psbB (with single intron IEP)]. The tree was constructed using a dataset of homologs retreived by BLASTp (e-value cutoff = 1e−5) from the NCBI non-redundant database using the F. japonica psbB group II intron IEP as a query. Numbers (Bootstrap values > 50%) on branches are IQ-Tree UFBoot. The scale bar shows the inferred number of amino acid substitutions per site. [file Data_Sheet_5.PDF]

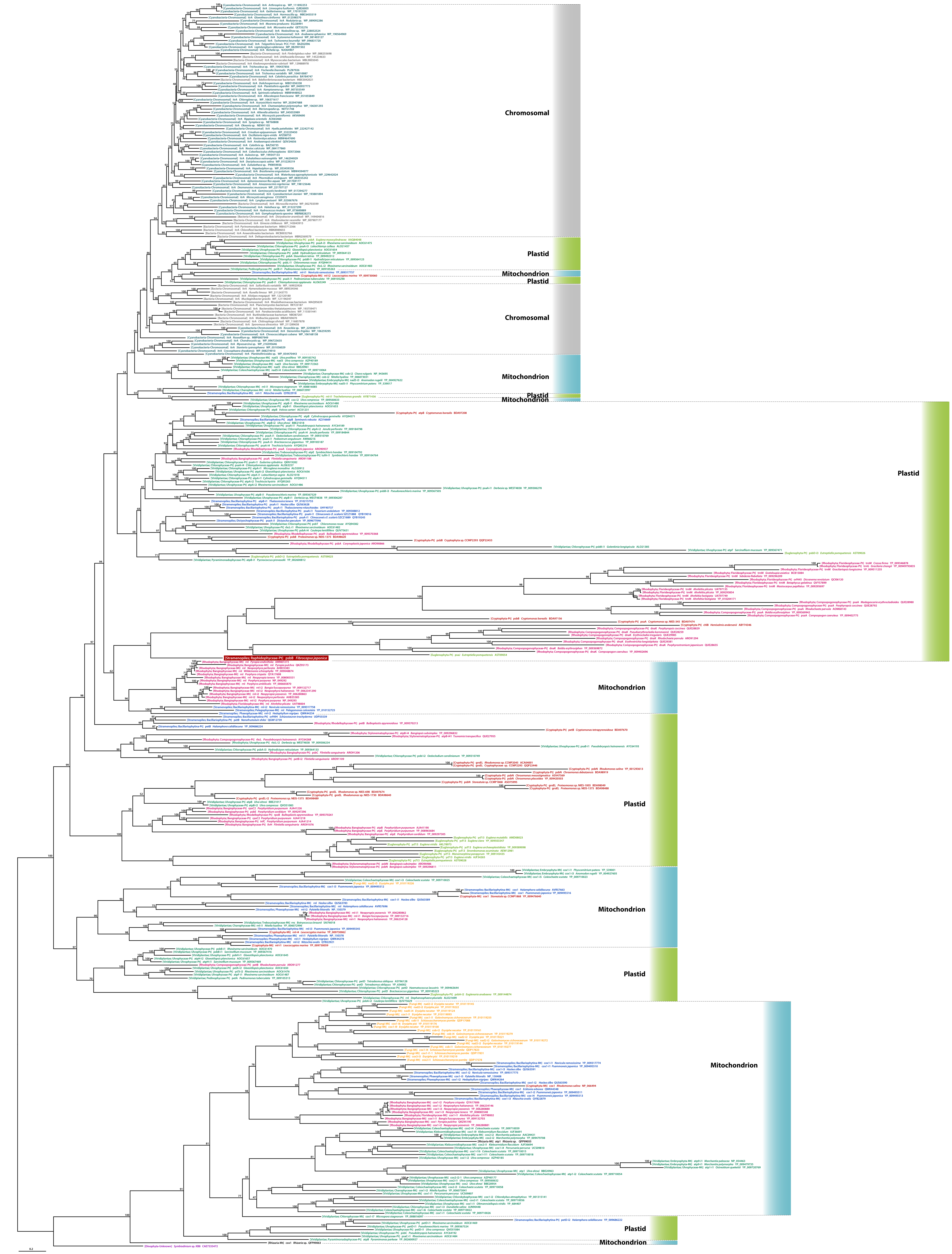
Chromosomal
Plastid
Mitochondrion
Plastid
Chromosomal
Mitochondrion
Plastid
Mitochondrion
Plastid
Mitochondrion
Plastid
Mitochondrion
Plastid
Mitochondrion
Plastid
Mitochondrion
0.2
